# Monocular Deprivation Affects Visual Cortex Plasticity Through cPKCγ-Modulated GluR1 Phosphorylation in Mice

**DOI:** 10.1167/iovs.61.4.44

**Published:** 2020-04-28

**Authors:** Yunxia Zhang, Tao Fu, Song Han, Yichao Ding, Jing Wang, Jiayin Zheng, Junfa Li

**Affiliations:** 1 Department of Neurobiology, School of Basic Medical Sciences, Capital Medical University, Beijing, China; 2 Beijing Tongren Eye Center, Beijing Tongren Hospital, Capital Medical University, Beijing Ophthalmology and Visual Sciences Key Laboratory, Beijing, China

**Keywords:** visual cortex plasticity, monocular deprivation, long-term potentiation, cPKCγ

## Abstract

**Purpose:**

To determine how visual cortex plasticity changes after monocular deprivation (MD) in mice and whether conventional protein kinase C gamma (cPKCγ) plays a role in visual cortex plasticity.

**Methods:**

cPKCγ membrane translocation levels were quantified by using immunoblotting to explore the effects of MD on cPKCγ activation. Electrophysiology was used to record field excitatory postsynaptic potential (fEPSP) amplitude with the goal of observing changes in visual cortex plasticity after MD. Immunoblotting was also used to determine the phosphorylation levels of GluR1 at Ser831. Light transmission was analyzed using electroretinography to examine the effects of MD and cPKCγ on mouse retinal function.

**Results:**

Membrane translocation levels of cPKCγ significantly increased in the contralateral visual cortex of MD mice compared to wild-type (WT) mice (*P* < 0.001). In the contralateral visual cortex, long-term potentiation (LTP) and the phosphorylation levels of GluR1 at Ser 831 were increased in cPKCγ^+/+^ mice after MD. Interestingly, these levels could be downregulated by *cPKCγ* knockout compared to cPKCγ^+/+^+MD mice (*P* < 0.001). Compared to the right eyes of WT mice, the amplitudes of a-waves and b-waves declined in deprived right eyes of mice after MD (*P* < 0.001). There were no significant differences when comparing cPKCγ^+/+^ and cPKCγ^−/−^ mice with MD.

**Conclusions:**

cPKCγ participates in the plasticity of the visual cortex after MD, which is characterized by increased LTP in the contralateral visual cortex, which may be a result of cPKCγ-mediated phosphorylation of GluR1 at Ser 831.

Synaptic plasticity is crucial to our responding flexibly to various environments by changing synaptic connections between neurons.^1^ Activity-dependent synaptic remodeling is a key mechanism mediating neural circuit adaptation and brain plasticity.^2^ When visual experience regulating visual cortical circuits was deprived, the plasticity of the visual cortex changed.^3^^,^^4^

In animals, monocular deprivation (MD), which was reported in 1963 to induce amblyopia,^5^ is often used to study visual cortex plasticity. Studies using mice have found that MD induces robust plasticity of the visual cortex during critical periods through degradation of the extracellular matrix by upregulation of proteases, thus elevating spine motility.^6^ Similar studies using P17 mice have shown that MD strengthens excitatory synaptic connections of layer 4^7^ and induces plasticity in layers 2 and 3 of the deprived cortex.^8^ However, some studies have shown that, after MD, miniature inhibitory postsynaptic currents (IPSCs) and the density of postsynaptic GABA_A_ receptors were increased in layer 4 of the visual cortex,^9^ indicating that the plasticity of the visual cortex was decreased after MD. Thus, how visual cortex plasticity changes after MD is incompletely understood.

Conventional protein kinase C gamma (cPKCγ), a member of the PKC family of Ser/Thr kinases that regulate a series of cellular processes, is specifically expressed in neurons, and cPKCγ may play a key role in synaptic plasticity. In mutant cPKCγ mice, cPKCγ membrane translocation kinetics are impaired, which may destroy synaptic plasticity, synapse pruning, and synaptic transmission.^10^ The offspring of male mice exposed to postnatal traumatic stress have decreased signaling components of cPKCγ in the hippocampus which damages synaptic plasticity when they reach adulthood.^11^ cPKCγ mediates post-tetanic potentiation (PTP) by increasing the probability of release in the auditory brainstem^12^ and maintains the function of Purkinje cells by regulating the phosphorylation and insertion of GABA_A_γ2 into the postsynaptic membrane.^13^ The activation of cPKCγ may mediate the neuroprotective effects of resveratrol and epigallocatechin gallate polyphenols on the cytoskeleton, as well as synaptic plasticity.^14^ In inflammation-induced mechanical allodynia, interneurons expressing cPKCγ are involved in strong morphological reorganization via 5-HT_2A_ receptor activation.^15^ However, few studies have investigated the roles of cPKCγ in visual cortex plasticity. In our previous work, *cPKCγ* knockout significantly changed the dynamic expression of P-synapsin-Ia/b at sites Ser603 and Ser9,^16^ which may play a role in the synaptic plasticity of the visual cortex.^17^ Here, we further explore whether cPKCγ participates in visual cortex plasticity in MD mice.

## Materials and Methods

### Animals and Monocular Deprivation

The C57BL/6J wild-type (WT, cPKCγ^+/+^) and *cPKCγ* knockout (KO, cPKCγ^−/−^) mice were purchased from The Jackson Laboratory (Bar Harbor, ME, USA). All animals were housed in a barrier system with constant temperature and humidity and a 12-hour light/dark cycle, and they were exposed to food and water. All procedures were performed according to the ARVO Statement for the Use of Animals in Ophthalmic and Vision Research and adhered to the guidelines required by Animal Care and Use Committee of Capital Medical University.

MD was implemented by eyelid suturing at postnatal day 7 (P7) to imitate amblyopia caused by congenital cataracts in mice. Animals were anesthetized by intraperitoneal (IP) injection of pentobarbital sodium (0.06 g/kg). Erythromycin eye ointment (Guangzhou Baiyunshan Pharmaceutical Co., Ltd., Guangzhou, China) was given to MD mice to prevent infection. Mice were then housed for P21 days.

Seventy-two male and female mice were randomly divided into four groups: cPKCγ^+/+^, cPKCγ^+/+^+MD (cPKCγ^+/+^ mice with MD), cPKCγ^−/−^, and cPKCγ^−/−^+MD (cPKCγ^−/−^ mice with MD). The visual cortexes of mice in the cPKCγ^+/+^ and MD groups were prepared to determine the membrane translocation of cPKCγ after electroretinography (n = 6 per group). The visual cortexes of mice in the four groups was prepared to determine GluR1 phosphorylation after electroretinography (n = 6 per group). Mice in the cPKCγ^+/+^ and cPKCγ^+/+^+MD groups were used for immunofluorescence staining (n = 6 per group). Mice in all four groups were also used for electrophysiology (n = 6 per group).

### Immunofluorescence

Mice at P21 days were anesthetized using 1% pentobarbital sodium (0.07 g/kg, IP injection) and perfused transcardially with 0.9% NaCl for 1 minute followed by 4% paraformaldehyde in 0.1-M PBS (pH 7.4) for 15 minutes. Brains were quickly removed, post-fixed in 4% paraformaldehyde at 4°C for 24 hours, and dehydrated individually in 20% and then 30% sucrose solutions at 4°C for 24 hours in each solution. Brains were embedded in optimal cutting temperature compound (ZLI-9302; Sakura Finetek Japan Co., Ltd., Tokyo, Japan) and frozen in liquid nitrogen for 5 minutes. Next, brains were frozen at −80°C for 24 hours. According to the Mouse Brain Atlas, regional location of the visual cortex was classified as bregma (–2.18 to –5.20 mm). Cryoprotected brains were sectioned into slices 20-µm thick and containing visual cortex in the coronal plane by using a microtome (CM1950 Clinical Cryostat; Leica Biosystems, Wetzlar, Germany).

For immunofluorescence staining, brain slices were perforated using PBS Triton X-100 buffer (0.5% Triton X-100 in 0.1-M PBS; Sigma-Aldrich, St. Louis, MO, USA) for 30 minutes and then incubated with 8% goat serum in PBS (0.1-M) for 1 hour at room temperature. Primary mouse antibodies against the neuron-specific marker NeuN (ab104224, 1:300; Abcam, Cambridge, UK) were added at 4°C overnight. Following washing with PBS six times for 10 minutes each, the slices were incubated in Alexa Fluor 488 goat anti-mouse IgG (A11029, 1:300; Thermo Fisher Scientific, Watham, MA, USA) secondary antibody for 2 hours at room temperature. Finally, 2-(4-amidinophenyl)-6-indolecarbamidine dihydrochloride containing mounting media was used to mount slices. Microphotographs were taken using a Leica microscope, and images of the visual cortex were taken for each slice. Image J software (National Institutes of Health, Bethesda, MD, USA) was used to blindly count the number of NeuN-staining positive cells per image in a blind manner.

### Immunoblotting

Brain tissue from all four groups and regions containing the visual cortex were collected. The mouse visual cortex was dissected and rapidly frozen in liquid nitrogen. Based on our previous work,^18^^,^^19^ frozen samples were thawed and homogenized in Buffer A (50-mM Tris-Cl, pH 7.5; 1-mM EGTA; 2 mM-EDTA; 100-µM sodium vanadate; 50-nM okadaic acid; 50-mM potassium fluoride; 5-mM sodium pyrophosphate; and 5 µg/µL each of pepstatin A, chymostatin, leupeptin, and aprotinin). Homogenates were then centrifuged at 100,000*g* for 30 minutes at 4°C, and the supernatants were collected as the cytosolic fraction. The pellets were resuspended, sonicated, and completely dissolved in Buffer C (Buffer A containing 2% SDS^20^) as the membrane fraction. The cytosolic and membrane fractions were used to investigate membrane translocation of cPKCγ, and the membrane fractions were used to analyze phosphorylated GluR1 (pGluR1) at Ser 831 levels. Protein concentrations were determined using a bicinchoninic acid kit (Pierce Biotechnology, Rockford, IL, USA). Albumin dissolved in Buffer A or C at various concentration was used as the standard.

Protein samples (10 µg/lane) were separated using 10% SDS-PAGE, and the proteins were then transferred onto polyvinylidene difluoride (PVDF) membranes (0.22 µm; GE Healthcare, Chicago, IL, USA). The transferred PVDF membrane was blocked in 10% no-fat milk in A Tween-20 (Sigma-Aldrich)/Tris-buffered salt solution (TTBS; 20-mM Tris-Cl, pH7.5; 0.15-M NaCl; and 0.05% Tween-20) for 1 hour at room temperature. After washing in TTBS three times for 10 minutes each, the membranes were incubated overnight at 4°C in primary antibodies such as anti-cPKCγ (sc-211, 1:1000; Santa Cruz Biotechnology, Inc., Dallas, TX, USA), anti-phospho-GluR1 (Ser831) (ab109464, 1:1000; Abcam), anti-GluR1 (ab109450, 1:1000; Abcam), anti-β-actin (60008-1-Ig, 1:10000; ProteinTech Group, Rosemont, IL, USA), and anti-Na-K-ATPase (ab76020, 1:1000; Abcam). Membranes were then rinsed with TTBS three times (10 minutes each), and incubated in goat anti-rabbit or anti-mouse IgG secondaries at 1:5000 dilutions for 1 hour at room temperature. After rinsing once more with TTBS, protein signal was detected using an enhanced chemiluminescent reagent solution (chemiluminescent horseradish peroxidase substrate; MilliporeSigma, Burlington, MA, USA) and Fusion FX (Vilber Lourmat, Marne-la-Vallée, France).

Quantitative analysis of immunoblots was performed using Fusion Capt 16.15 software (Fusion FX6 XT; Vilber Lourmat). The ratio of the band density of membrane to the band density of the corresponding Na^+^-K^+^-ATPase and the ratio of the band density of cytosol to the band density of the corresponding β-actin were calculated. Finally, cPKCγ membrane translocation levels were expressed as the ratio of band density in the membrane fraction to the band densities in both cytosolic and membrane fractions. The pGluR1 (Ser831) levels were expressed as the ratio of the band density in phospho-GluR1 (Ser831) to band density in GluR1.

### Electrophysiology

At P21, the mice were anesthetized using 1% pentobarbital sodium (0.07g/kg, IP) and perfused transcardially with 10 mL ice-chilled dissection slicing solution, which contained the following: 213-mM sucrose, 10-mM d-glucose, 1-mM NaH_2_PO_4_, 3-mM KCl, 26-mM NaHCO_3_, 0.5-mM CaCl_2_, and 5-mM MgCl_2_ (pH 7.4). Brains were then quickly removed and trimmed in ice-chilled dissection slicing solution. Visual cortical slices (400 µm) were cut with a microslicer (DTK-1000; DSK, Kyoto, Japan) and recovered in artificial cerebral spinal fluid (ACSF) for at least 1 hour at room temperature. The ACSF contained the following: 126-mM NaCl, 25-mM d-glucose, 25-mM NaHCO_3_, 1-mM NaH_2_PO_4_, 3.5-mM KCl, 2-mM CaCl_2_, and 1-mM MgSO_4_, (pH 7.4), equilibrated with 95% O_2_ and 5% CO_2_. Before recording, the slices were transferred to a recording chamber and continuously perfused with ACSF.

Traces in the second and third layer (L2/3) of the visual cortex were recorded by stimulating the fourth layer (L4). They were obtained and analyzed using MED64 System software (Alpha MED Scientific, Inc., Osaka, Japan). To determine the most suitable current for each slice, the input-output (I-O) curve was first recorded by the measurements of field excitatory postsynaptic potential (fEPSP) amplitude. The current corresponding to 30% to 50% of the maximum value of the I-O curve was recognized as the most suitable. The slices were stimulated at the most suitable current one time every minute. After a baseline was recorded for additional 10 minutes, long-term potentiation (LTP) was induced by high-frequency stimulation (HFS) composed of two 1-second trains of 100-Hz pulses, with a 60-second interval.^21^ Responses were recorded for 70 minutes after HFS induction.

### Electroretinography

Before ERG testing, the mice were dark-adapted overnight and anesthetized with pentobarbital sodium (0.06 g/kg, IP). Pupils were dilated using tropicamide phenylephrine eye drops (Santen Pharmaceutical Co., Ltd., Osaka, Japan). Corneal surfaces were anesthetized with 0.5% proparacaine hydrochloride eye drops (Alcaine; Alcon, Geneva, Switzerland) and were covered with carbomer eye drops (Gerhard Mann, Chem Pharm Fabrik Gmbh) to increase conductivity and prevent drying. Espion Red and Espion v6 (Diagnosys, Lowell, MA, USA) were used to test ERGs. In dark-adapted ERGs, the flash luminance was 0.01 cd·s/m and 20 cd·s/m. In light-adapted ERGs after 5 minutes of light adaptation, the flash luminance was 20 cd·s/m.

### Statistical Analysis

All data are presented as mean ± SEM. The number of NeuN^+^ cells in visual cortex of mice was analyzed with three-way repeated ANOVA. Other statistical analysis was performed using two-way repeated ANOVA and followed pairwise multiple comparisons using Bonferroni if the interaction effect was statistically significant based on GraphPad Prism 6 software (GraphPad, San Diego, CA, USA). *P* < 0.05 was considered statistically significant.

## Results

### Effects of MD on the Number of Neurons and cPKCγ Membrane Translocation Levels in the Visual Cortex

**Figure 1.**
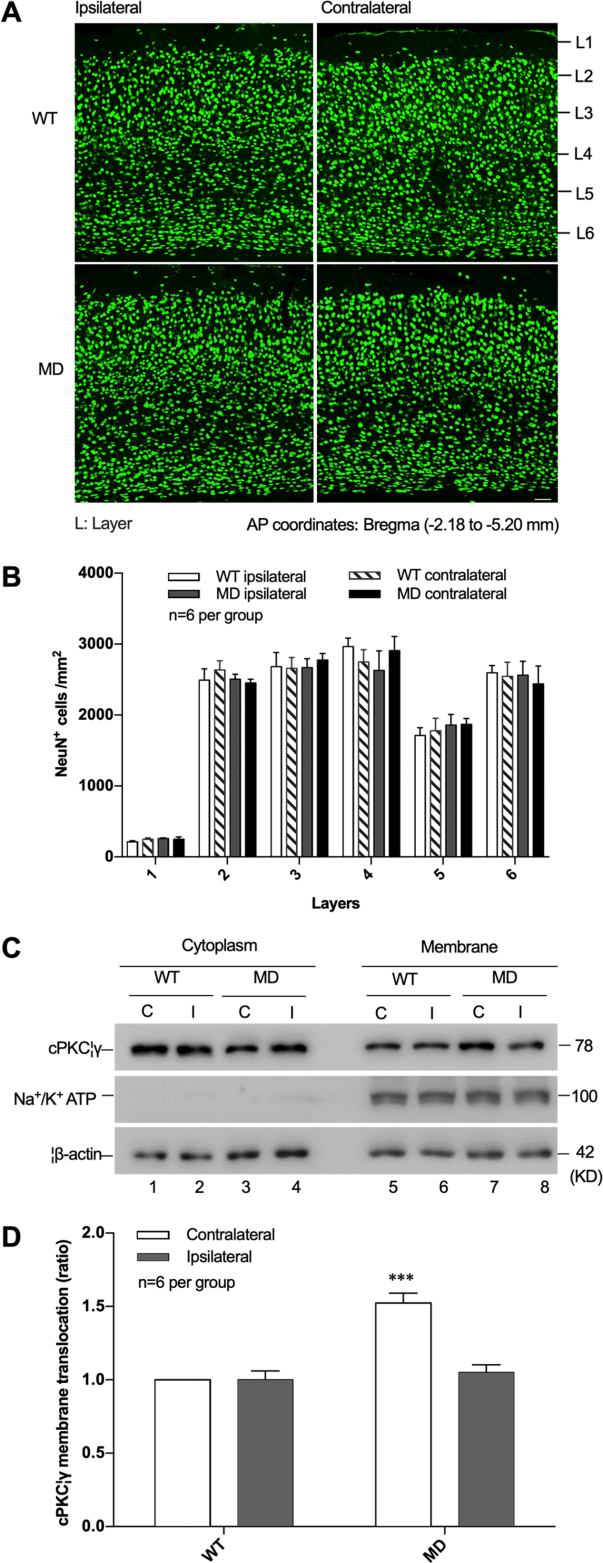
Effects of MD on the number of neurons and cPKCγ membrane translocation levels in the visual cortex. (**A**) Representative images of visual cortical immunostaining for NeuN^+^ cells in WT and MD mice. *Scale bar*: 50 µm. (**B**) Statistical results of NeuN^+^ cells in visual cortex of WT and MD mice. (**C**) Representative western blot images show the membrane translocation of cPKCγ in the visual cortex of WT and MD mice. (**D**) Statistical results of western blot analysis for the membrane translocation levels of cPKCγ in the visual cortex of WT and MD mice. ^***^P < 0.001 compared with the WT contralateral mice group (n = 6 per group).

First, we wanted to determine whether MD affected neuron numbers and cPKCγ membrane translocation levels in the visual cortex. As shown in Figures 1A and 1B, the number of NeuN-staining positive cells did not change in each layer of the visual cortex after MD, which indicated that MD did not affect neuron numbers in each visual cortex layer (Fig. 1B) after taking out the factors of layer, side (ipsilateral/contralateral), and genotype. Furthermore, the ratio of cPKCγ in membrane to cytosol was significantly increased in the contralateral visual cortex by 52% after MD: *F*(1, 20) = 22.0622, *P* < 0.001, and n = 6 per group (Figs. 1C, 1D). However, no significant changes in total cPKCγ protein expression levels were observed in either the contralateral or ipsilateral visual cortex of MD mice.

### Role of cPKCγ in the MD-Induced Increase of LTP in the Contralateral Visual Cortex

**Figure 2.**
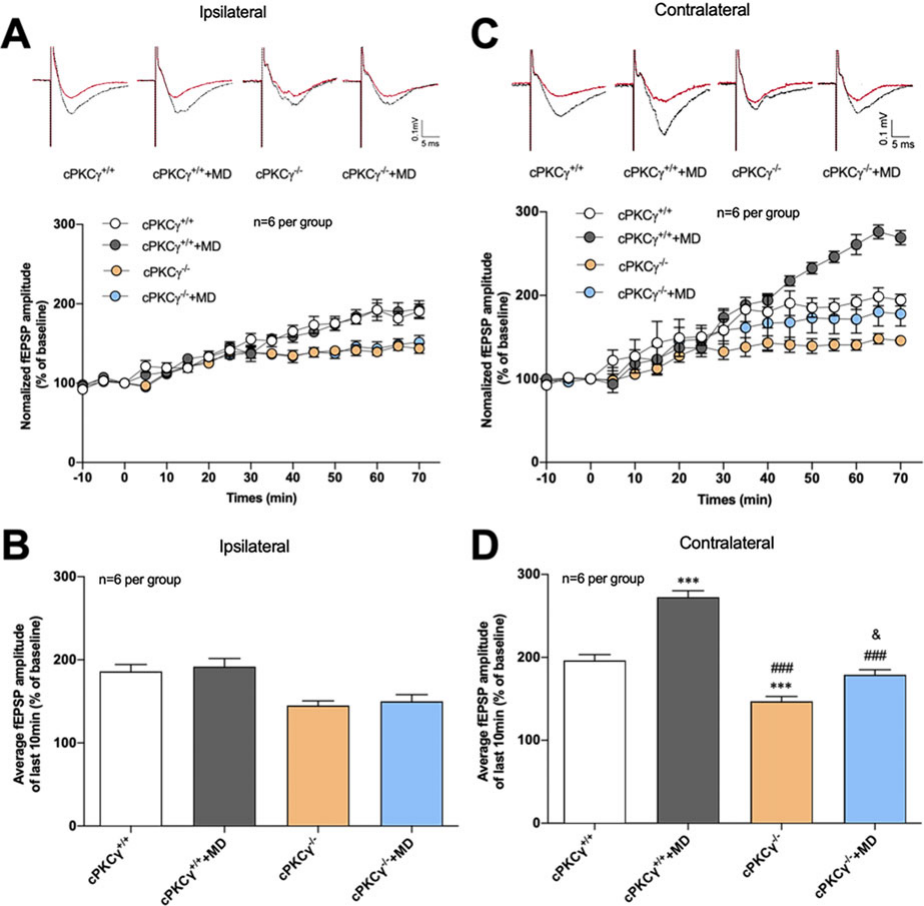
Role of cPKCγ in the MD-induced elevation of LTP in contralateral visual cortex. (**A**) Effects of MD and cPKCγ on the amplitude of fEPSPs recorded in ipsilateral visual cortex. (**B**) LTP levels recorded 60 to 70 minutes after HFS in ipsilateral visual cortex (mean ± SEM). (**C**) Effects of MD and cPKCγ on the amplitudes of fEPSPs recorded in contralateral visual cortex. (**D**) LTP levels recorded 60 to 70 minutes after HFS in contralateral visual cortex (mean ± SEM). HFS was composed of two 1-second trains of 100-Hz pulses with a 60-second interval. The recording and stimulating electrodes were located in L4 and L2/3 of the visual cortex, respectively. ^***^P < 0.001 compared with the cPKCγ^+/+^ mice group; ^###^*P* < 0.001 compared with the cPKCγ^+/+^+MD mice group; ^&^*P* < 0.05 compared with the cPKCγ^−/−^ mice group (n = 6 per group).

We have shown that synapsin-Ia/b is involved in visual cortex development^17^ and that cPKCγ can modulate the phosphorylation status of synapsin-Ia/b.^16^ We also wanted to determine the role of cPKCγ in visual cortex plasticity after MD. To examine the effects of MD on visual cortical LTP in cPKCγ^+/+^ and cPKCγ^−/−^ mice, we conducted field visual cortical LTP recordings and determined I-O curves. Results revealed no differences in I-O curves among the four groups (Supplementary Figs. S1A, S1B; Table S1), indicating that there are no differences in basal transmission among the different experimental groups. As shown in Figures 2A and 2B, MD did not cause a change in LTP in the ipsilateral visual cortexes of cPKCγ^+/+^ and cPKCγ^−/^^−^ mice (Fig. 2B); cPKCγ^+/+^, cPKCγ^+/+^+MD, cPKCγ^−/−^, and cPKCγ^−/−^+MD were 186.119 ± 8.302, 191.780 ± 9.764, 145.028 ± 5.615, and 150.130 ± 7.929, respectively. In addition, the amplitude of fEPSP in cPKCγ^−/−^ mice was decreased (*P* < 0.01) compared to cPKCγ^+/+^ mice—*F*(1, 20) = 26.4659, *P* < 0.001, n = 6 per group—indicating that *cPKCγ* knockout resulted in a decrease in LTP. In the contralateral visual cortex (Figs. 2C, 2D), MD caused a 76% increase in the LTP in cPKCγ^+/+^ mice (*P* < 0.001), but only a 32% increase (*P* < 0.05) was observed in the LTP of cPKCγ^−/−^ mice: *F*(1, 20) = 10.6467, *P* = 0.004, and n = 6 per group. This finding indicated that LTP was significantly increased in the contralateral visual cortex of cPKCγ^+/+^+MD mice compared to cPKCγ^−/−^+MD mice (*P* < 0.001, n = 6 per group), which means that a cPKCγ deficiency could reduce the elevation of LTP induced by MD.

### Role of cPKCγ in the MD-Induced Increase of pGluR1 (Ser831) Levels in the Contralateral Visual Cortex

**Figure 3.**
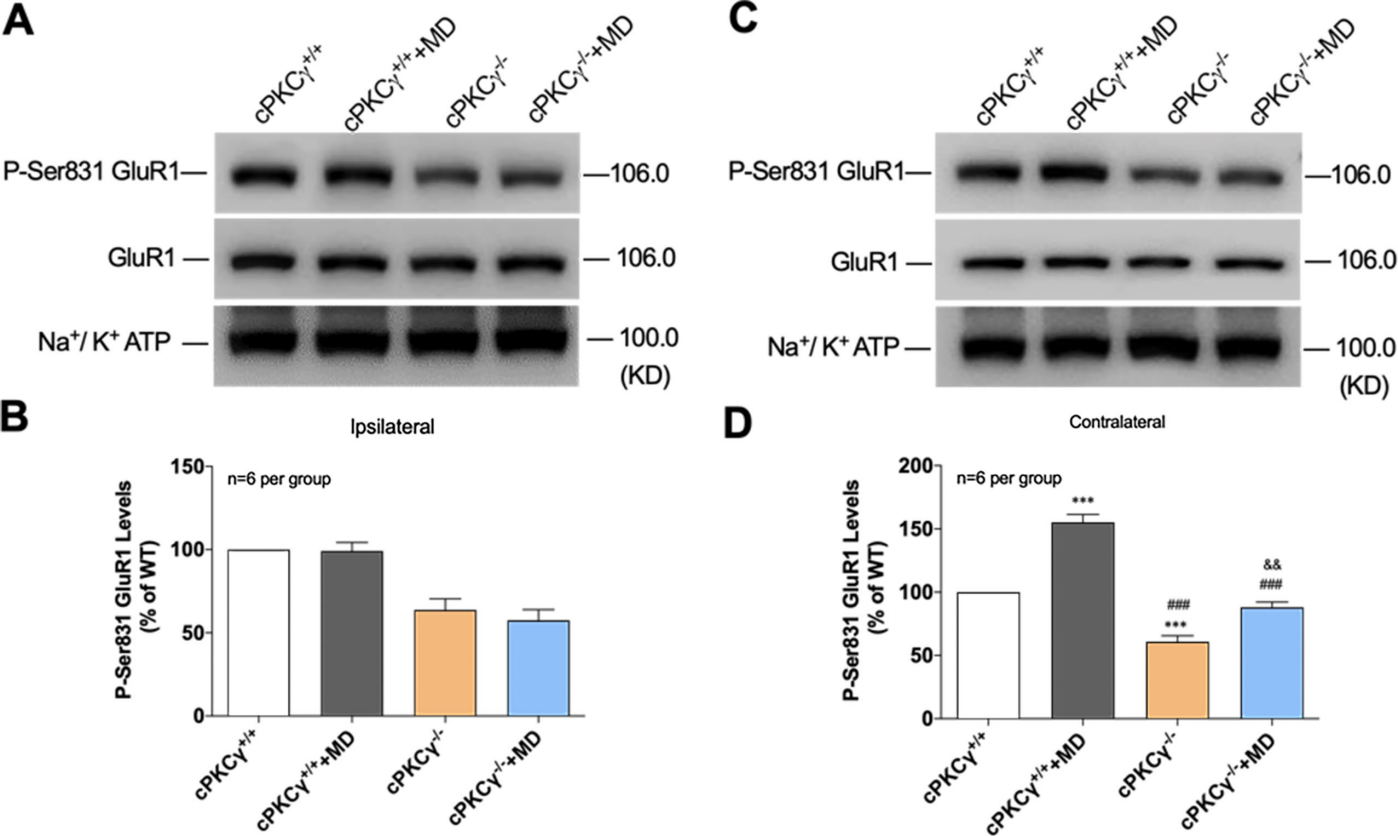
Role of cPKCγ in the MD-induced increase of phosphorylation levels of GluR1 at Ser831 in contralateral visual cortex. (**A**) Representative western blot images show the pGluR1 (Ser831) levels in ipsilateral visual cortex from cPKCγ^+/+^ and cPKCγ^−/−^ mice after MD. (**B**) Statistical results of western blot analysis demonstrate pGluR1 (Ser831) levels in ipsilateral visual cortex of cPKCγ^+/+^ and cPKCγ^−/−^ mice after MD. (**C**) Representative western blot images show pGluR1 (Ser831) levels in contralateral visual cortex from cPKCγ^+/+^ and cPKCγ^−/−^ mice after MD. (**D**) Statistical results of western blot analysis show the changes of pGluR1 (Ser831) levels in contralateral visual cortex of cPKCγ^+/+^ and cPKCγ^−/−^ mice after MD. ^***^P < 0.001 compared with the cPKCγ^+/+^ mice group; ^###^*P* < 0.001 compared with the cPKCγ^+/+^+MD group; ^&&^*P* < 0.01 compared with the cPKCγ^−/−^ mice group (n = 6 per group).

To analyze the possible molecular mechanisms of cPKCγ in the MD-induced increase of LTP in the contralateral visual cortex, pGluR1 levels at Ser831 (P-Ser831 GluR1) were examined. GluR1 is a subunit of the α-amino-3-hydroxy-5-methyl-4-isoxazolepropionic acid (AMPA) receptor, which consists of four subunits, including GluR1-4, and mediates rapid excitatory synaptic transmission in the central nervous system.^22^ Furthermore, the AMPA receptor is associated with the induction and maintenance of LTP.^23^ As shown in Figures 3A and 3B, MD did not cause a change in pGluR1 levels at Ser831 in the ipsilateral visual cortex of cPKCγ^+/+^ and cPKCγ^−/−^ mice; cPKCγ^+/+^, cPKCγ^+/+^+MD, cPKCγ^−/−^, and cPKCγ^−/−^+MD mice were 100.000 ± 0.000, 99.130 ± 5.126, 63.813 ± 6.681, and 57.595 ± 6.292, respectively (Fig. 3B). In addition, compared to cPKCγ^+/+^ mice, pGluR1 levels at Ser831 were significantly (*P* < 0.001) decreased in cPKCγ^−/^^−^ mice, indicating that *cPKCγ* knockout resulted in decreased pGluR1 levels at Ser831: *F*(1, 20) = 54.6622, *p* < 0.001, and n = 6 per group. In cPKCγ^+/+^ and cPKCγ^−/−^ mice, MD increased pGluR1 levels at Ser831 in the contralateral visual cortex by 55% (*P* < 0.001) and 27% (*P* < 0.01), respectively: *F*(1, 20) = 10.0530, *P* = 0.005, and n = 6 per group (Figs. 3C, 3D). This indicated that cPKCγ deficiency could reduce the elevated pGluR1 levels at Ser831 that were induced by MD in the contralateral visual cortex.

### Effects of MD and cPKCγ on Light Detection and Transmission

**Figure 4.**
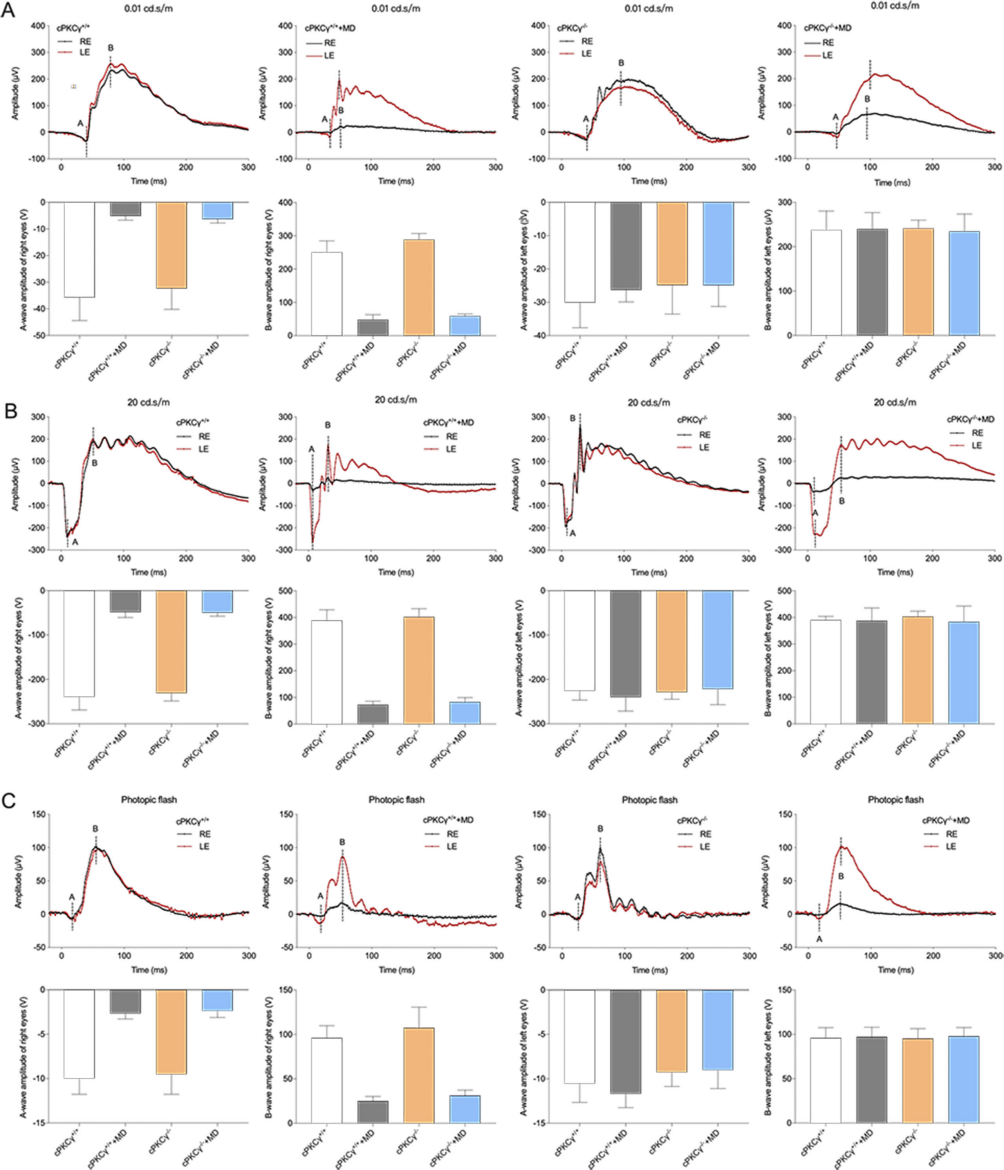
Effects of MD and cPKCγ on light detection and transmission. (**A**) Typical dark-adapted ERG waveforms and statistical results of a-wave and b-wave amplitudes at a flash intensity of 0.01 cd·s/m in cPKCγ^+/+^ and cPKCγ^−/−^ mice after MD. (**B**) Typical dark-adapted ERG waveforms and statistical results of a-wave and b-wave amplitudes at a flash intensity of 20 cd·s/m in cPKCγ^+/+^ and cPKCγ^−/−^ mice after MD. (**C**) Typical light-adapted ERG waveforms and statistical results of a-wave and b-wave amplitudes at a flash intensity of 20 cd·s/m in cPKCγ^+/+^ and cPKCγ^−/−^ mice after MD (n = 6 per group).

Retinal function of light transmission was tested using ERG typically composed of a-waves and b-waves. In dark-adapted ERG at a flash intensity of 0.01 cd·s/m, the a-wave is the reaction from rod photoreceptors, and the b-wave is from bipolar cells of the rod pathways.^24^ As shown in Figure 4A, the amplitudes of the a-waves—*F*(1, 20) = 22.8871, *P* < 0.001—and the amplitudes of the b-waves—*F*(1, 20) = 150.240, *P* < 0.001—in the right eye dropped after MD in dark-adapted ERG at a flash intensity of 0.01 cd·s/m (n = 6 per group) (Fig. 4A), suggesting that transmission of the rod system was affected by MD. There were no differences observed in the amplitudes of the a-waves and b-waves of the right eye when comparing cPKCγ^+/+^ and cPKCγ^−/^^−^ mice with MD. This indicates that cPKCγ does not affect transmission of the rod system.

In dark-adapted ERG at a flash intensity of 20 cd·s/m, the a-wave is produced by rod and cone photoreceptors, and the b-wave is mainly derived from the bipolar cell of the cone and rod.^25^ We observed a significant drop of a-wave amplitudes—*F*(1, 20) = 98.2604, *P* < 0.001—and b-wave amplitudes—*F*(1, 20) = 136.106, *P* < 0.001—in the right eyes of mice after MD in dark-adapted ERG at a flash intensity of 20 cd·s/m (n = 6 per group) (Fig. 4B). Similarly, there were no differences between cPKCγ^+/+^ and cPKCγ^−/^^−^ mice with MD: a-wave, –48.595 ± 12.068 versus –49.907 ± 7.780; b-wave, 72.522 ± 12.969 versus 83.245 ± 16.255 (n = 6 per group) (Fig. 4B).

In light-adapted ERG, the a-wave is generated by cone photoreceptors, and the b-wave is generated from bipolar cells of the cone pathways.^24^ As shown in Figure 4C, the a-wave amplitudes—*F*(1, 20) = 22.3795, *P* < 0.001—and b-wave amplitudes—*F*(1, 20) = 27.6757, *P* < 0.001—were reduced in light-adapted ERG in the right eye of MD mice (n = 6 per group), indicating that transmission of the cone system was affected by MD. No notable differences in the amplitudes of the a-wave or b-wave were observed in the right eye when comparing cPKCγ^+/+^ and cPKCγ^−/−^ mice with MD (Fig. 4C), indicating that cPKCγ did not affect transmission of the cone system.

## Discussion

In this study, we investigated the role of cPKCγ in visual cortex plasticity using a C57BL/6J MD mouse model. We used slice electrophysiology to show that LTP in the contralateral visual cortex of the deprived eye increased significantly. Through the use of immunoblotting, we determined that the level of cPKCγ membrane translocation in the contralateral visual cortex was upregulated. Knockout of *cPKCγ* reduced phosphorylation levels of the AMPA receptor GluR1 subunit at Ser831 and downregulated the elevated levels of LTP. Light transmission was impaired in the deprived eye by ERG. It was determined that cPKCγ did not play a role in these changes after MD. Based on these results, it can be inferred that cPKCγ contributes to visual cortex plasticity after MD through the phosphorylation of the AMPA receptor GluR1 subunit at Ser831.

cPKCγ is specifically distributed in neurons and found mainly in the cytoplasm. When neurons are stimulated, cPKCγ translocates from the cytoplasm to the cell membrane in a Ca^2+^-dependent manner.^26^ The phenomenon of cPKCγ membrane translocation is generally considered to be a marker for cPKCγ activation. Activated cPKCγ can bind to and phosphorylate other proteins in the cell, regulating biochemical reactions.^27^ In previous studies, it was found that cPKCγ activation was involved in many pathophysiological processes. In mice enduring cerebral ischemic injuries, cPKCγ was activated and worked to protect cortical neurons.^28^ In addition, hypoxic preconditioning induced cPKCγ activation and also protected mice from cerebral ischemic injuries through the phosphorylation of synapsin.^29^ The membrane translocation levels of cPKCγ have been shown to decrease in the cortex and striatum of mice with diabetic encephalopathy.^19^ In this study, we found that the levels of cPKCγ membrane translocation increased after MD, and neuron numbers did not change significantly in the deprived visual cortex, suggesting that increased cPKCγ activation levels were not induced by neuron numbers.

During development of the mouse visual cortex, binocular responses are created and the topographic map is both formed and refined before the eyes are opened. After the eyes are opened, orientation selectivity and ocular dominance develop. P21 to P35 is known as the critical period for ocular dominance (OD) plasticity.^4^^,^^30^ To better understand the neuroplasticity of binocular neurons in the primary visual cortex (V1), OD plasticity after MD has been studied extensively.^31^^,^^32^ MD shifted the OD away from the deprived eye and to the open eye, resulting in a reduction in visual acuity of the deprived eye. OD measures the responsiveness of the neuron to the stimulus, as it collects the reactivity of the corresponding cortical neurons after the visual information is transmitted from the retina.^33^^,^^34^ As MD impaired the light transmission of deprived eyes at the beginning of critical period (Fig. 4), slice electrophysiology was used to record the phenomenon of LTP, measuring the ability of neurons to change after they are stimulated. An advantage of slice electrophysiology is that stimulation does not pass through the retina but reaches the V1 directly. Therefore, LTP should be used to clarify the effects of MD on the plasticity of the corresponding visual cortex, especially the monocular area of the deprived cortex, which accepts information only from the deprived eye. In this study, we found that the fEPSP amplitude of the deprived visual cortex was increased in cPKCγ^+/+^ and cPKCγ^−/−^ mice after MD, suggesting that synapse functions may have changed. This finding is consistent with those of other studies.^23^ Sammons et al.^8^ found that LTP and long-term depression were higher in deprived versus control cortexes, indicating that MD increases plasticity in the deprived cortex. Furthermore, LTP was increased in the contralateral visual cortex by 76% after MD in cPKCγ^+/+^ mice but increased by only 32% in cPKCγ^−/−^ mice. This indicates that cPKCγ deficiency could reduce the elevation of LTP induced by MD in contralateral visual cortex and cPKCγ may take part in visual cortex plasticity.

AMPA receptors consist of four subunits: GluR1 to GluR4. The phosphorylation of GluR1 is required for synaptic plasticity and spatial memory.^35^^–^^37^ Recent studies have shown that cPKCγ induces phosphorylation of the GluR1 subunit at Ser831, affecting the Ca^2+^ permeability of the AMPA receptor and leading to increased excitability of neurons in a pain model.^38^^,^^39^ In this study, we found that the level of pGluR1 at Ser 831 was increased in the contralateral visual cortex by 55% after MD in cPKCγ^+/+^ mice; however, the level of pGluR1 at Ser 831 increased by only 27% in cPKCγ^−/−^ mice. These results suggest that *cPKCγ* knockout might reduce visual cortex plasticity induced by MD by downregulating the levels of the pGluR1 subunit at Ser831. Future studies should work to inhibit pGluR1 (Ser 831) and observe whether it can reduce LTP formation, to further confirm whether this mechanism contributes to visual cortex plasticity after MD. The fact that *cPKCγ* knockout could reduce but not fully suppress LTP and pGluR1 (Ser831) levels after MD in the contralateral visual cortex indicates that not only cPKCγ but also other forms of PKC may participate in visual cortex plasticity. A great deal of work has revealed that cPKCα and cPKCβ play a role in PTP and hippocampal learning and memory.^12^^,^^40^^,^^41^ It is necessary to verify whether cPKCα and cPKCβ play a role in visual cortex plasticity.

Visual pathways include the transmission of light to the retina, projection to the lateral geniculate body, and projection to the primary visual cortex. We examined the effects of MD and cPKCγ on light transmission of mice by ERG. We found that the amplitudes of the a-waves and b-waves in the right eye decreased after MD in all of the ERG examination conditions, indicating that light transmission of the deprived eye was impaired, which supports prior work.^42^^,^^43^ When comparing cPKCγ^+/+^+MD mice and cPKCγ^−/^^−^+MD mice, the amplitudes of the a-waves and b-waves in the right eye were not statistically different, indicating that cPKCγ did not affect light transmission of retina. This phenomena might be due to the fact that other subtypes of PKC play an important role in retina function, such as aPKCζ,^44^^,^^45^ nPKCδ,^44^^,^^46^ and cPKCα.^47^^,^^48^ In addition, the intraocular pressures of the MD and control eyes were 8.67 ± 0.380 mm Hg and 8.5 ± 0.365 mm Hg, respectively (n = 6 per group; *t* = 0.316; degrees of freedom = 10; *P* = 0.758). Other reasons for the drop in ERG amplitudes might be temporary impairment of retinal function,^42^ as well as the reduction of ganglion, inner nuclear, and outer nuclear cells^49^; axial elongation of eye^50^; and ischemic insult^51^ in the deprived eye. We will examine the specific cause of ERG amplitude reduction in future studies.

In summary, the plasticity of the deprived cortex was higher after MD, although light transmission was impaired in the deprived eye. cPKCγ may participate in the plasticity of the visual cortex after MD through phosphorylation of GluR1 at Ser 831. In addition, a cPKCγ agonist might be used to improve visual cortex plasticity and assist in restoring visual function.

## Supplementary Material

Supplement 1

Supplement 2
