# Supplementary figures and images for: Monocular Deprivation Affects Visual Cortex Plasticity Through cPKCγ-Modulated GluR1 Phosphorylation in Mice
Source: Invest Ophthalmol Vis Sci. 2020 Apr 28;61(4):44. doi: 10.1167/iovs.61.4.44 (PMC7401946; doi:10.1167/iovs.61.4.44)

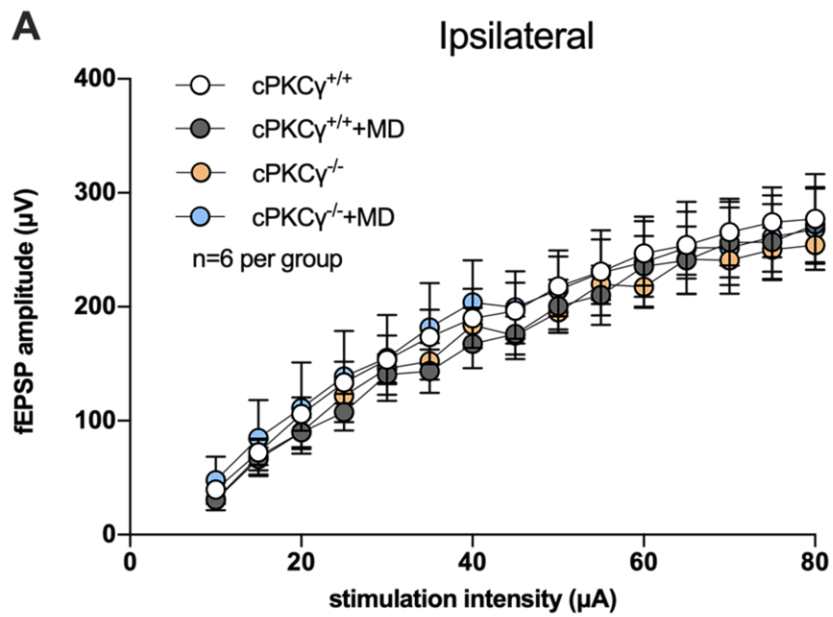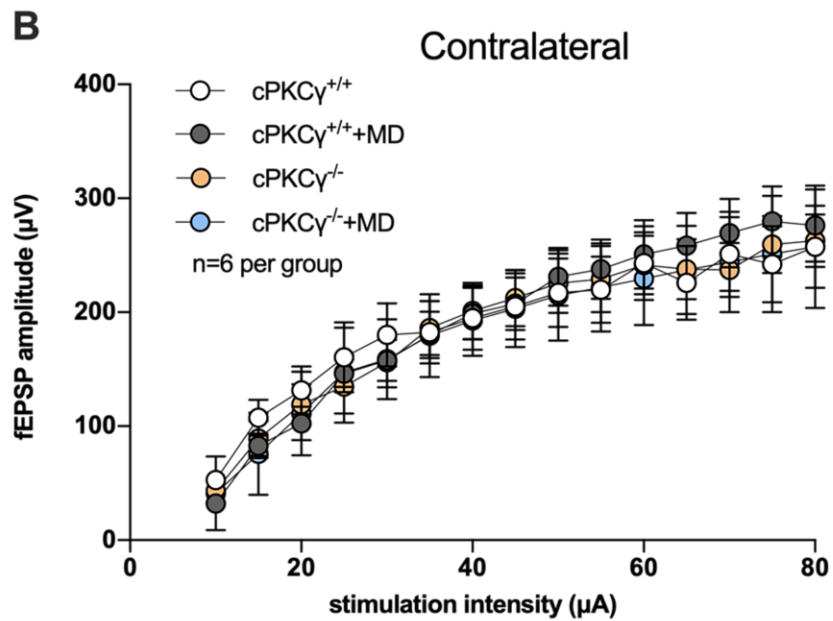

Supplement: Supplement 1 [file iovs-61-4-44_s001.pdf]
