# Supplementary material for: Monocular Deprivation Affects Visual Cortex Plasticity Through cPKCγ-Modulated GluR1 Phosphorylation in Mice
Source: Invest Ophthalmol Vis Sci. 2020 Apr 28;61(4):44. doi: 10.1167/iovs.61.4.44 (PMC7401946; doi:10.1167/iovs.61.4.44)
Supplement: Supplement 2 [file iovs-61-4-44_s002.pdf]

Supplement Table1. Two-way ANOVA of supplement Fig.1A and B

		Fig.1A (ipsilateral)	Fig.1B (contralateral)
10μA	Interaction	F (1, 20) = 0.2908,*p*=0.104	F (1, 20) = 0.9477,*p*=0.342
	WT/MD factor	F (1, 20) = 0.2499, *p*=0.263	F (1, 20) = 1.4613, *p*=0.241
	Genotype factor	F (1, 20) = 0.3715, *p*=0.549	F (1, 20) = 0.0010, *p*=0.975
15μA	Interaction	F (1, 20) = 0.6999,*p*=0.413	F (1, 20) =0.1653, *p*= 0.689
	WT/MD factor	F (1, 20) = 0.1684, *p*=0.686	F (1, 20) =1.7788, *p*=0.197
	Genotype factor	F (1, 20) = 0.3319, *p*=0.571	F (1, 20) =0.7948, *p*=0.383
20μA	Interaction	F (1, 20) = 1.5607,*p*=0.226	F (1, 20) =0.3753, *p*=0.547
	WT/MD factor	F (1, 20) = 0.0281, *p*=0.868	F (1, 20) =1.1154, *p*=0.304
	Genotype factor	F (1, 20) = 0.0408, *p*=0.842	F (1, 20) =0.0098, *p*=0.922
25μA	Interaction	F (1, 20) = 1.7373,*p*=0.203	F (1, 20) =0.3904, *p*=0.539
	WT/MD factor	F (1, 20) = 0.0742, *p*=0.788	F (1, 20) =0.0022, *p*= 0.963
	Genotype factor	F (1, 20) = 0.3651, *p*=0.552	F (1, 20) =0.3335, *p*=0.570
30μA	Interaction	F (1, 20) =0.4357, *p*=0.517	F (1, 20) =0.3546, *p*=0.558
	WT/MD factor	F (1, 20) =0.0102, *p*=0.920	F (1, 20) =0.3315, *p*=0.643
	Genotype factor	F (1, 20) =0.0433, *p*=0.837	F (1, 20) =0.2893, *p*=0.597
35μA	Interaction	F (1, 20) =2.3944, *p*=0.137	F (1, 20) =0.0094, *p*=0.924
	WT/MD factor	F (1, 20) =0.0000, *p*=0.996	F (1, 20) =0.0394, *p*=0.845
	Genotype factor	F (1, 20) =0.1995, *p*=0.660	F (1, 20) =0.0048, *p*=0.945
40μA	Interaction	F (1, 20) =1.1014, *p*=0.306	F (1, 20) =0.0728, *p*=0.790
	WT/MD factor	F (1, 20) =0.0031, *p*= 0.956	F (1, 20) =0.0098, *p*=0.922
	Genotype factor	F (1, 20) =0.5706, *p*=0.459	F (1, 20) =0.0001, *p*=0.993
45μA	Interaction	F (1, 20) =1.3115, *p*=0.266	F (1, 20) =0.0621, *p*=0.806
	WT/MD factor	F (1, 20) =0.0142, *p*=0.906	F (1, 20) =0.0234, *p*=0.880
	Genotype factor	F (1, 20) =0.0017, *p*=0.967	F (1, 20) =0.0032, *p*= 0.995
50μA	Interaction	F (1, 20) =0.7757, *p*=0.389	F (1, 20) =0.2430, *p*=0.627
	WT/MD factor	F (1, 20) =0.0034, *p*=0.954	F (1, 20) =0.0037, *p*=0.952
	Genotype factor	F (1, 20) =0.0433, *p*= 0.837	F (1, 20) =0.0252, *p*=0.875
55μA	Interaction	F (1, 20) =0.4975, *p*=0.489	F (1, 20) =0.2439, *p*=0.627
	WT/MD factor	F (1, 20) =0.0597, *p*=0.809	F (1, 20) =0.0429, *p*=0.838
	Genotype factor	F (1, 20) =0.0373, *p*=0.849	F (1, 20) =0.0183, *p*=0.894
60μA	Interaction	F (1, 20) =0.5223, *p*=0.478	F (1, 20) =0.1352, *p*=0.717
	WT/MD factor	F (1, 20) =0.0504, *p*=0.825	F (1, 20) =0.0063, *p*=0.937
	Genotype factor	F (1, 20) =0.3215, *p*=0.577	F (1, 20) =0.1834, *p*=0.673

65μA	Interaction	F (1, 20) =0.2388, *p*= 0.630	F (1, 20) =0.3980, *p*=0.535
	WT/MD factor	F (1, 20) =0.0054, *p*=0.942	F (1, 20) =0.3603, *p*=0.555
	Genotype factor	F (1, 20) =0.0009, *p*=0.976	F (1, 20) =0.0323, *p*=0.859
70μA	Interaction	F (1, 20) =0.1565, *p*=0.697	F (1, 20) =0.0460, *p*=0.832
	WT/MD factor	F (1, 20) =0.0016, *p*=0.968	F (1, 20) =0.2329, *p*=0.635
	Genotype factor	F (1, 20) =0.3426, *p*=0.565	F (1, 20) =0.5520, *p*=0.466
75μA	Interaction	F (1, 20) =0.3046, *p*=0.587	F (1, 20) =0.6702, *p*=0.423
	WT/MD factor	F (1, 20) =0.0159, *p*=0.901	F (1, 20) =0.2707, *p*=0.609
	Genotype factor	F (1, 20) =0.1492, *p*=0.703	F (1, 20) =0.4301, *p*=0.838
80μA	Interaction	F (1, 20) =0.1090, *p*=0.745	F (1, 20) =0.1746, *p*=0.681
	WT/MD factor	F (1, 20) =0.0239, *p*=0.879	F (1, 20) =0.0546, *p*=0.818
	Genotype factor	F (1, 20) =0.2149, *p*=0.648	F (1, 20) =0.0546, *p*=0.818
